# Benchmarking Periodic Density Functional Theory Calculations for Spin-State Energies in Spin-Crossover Systems

**DOI:** 10.1021/acs.inorgchem.4c01094

**Published:** 2024-07-08

**Authors:** Silvia Gómez-Coca, Eliseo Ruiz

**Affiliations:** Departament de Química Inorgànica i Orgànica and Institut de Recerca de Química Teòrica i Computacional, Universitat de Barcelona, Barcelona 08028, Spain

## Abstract

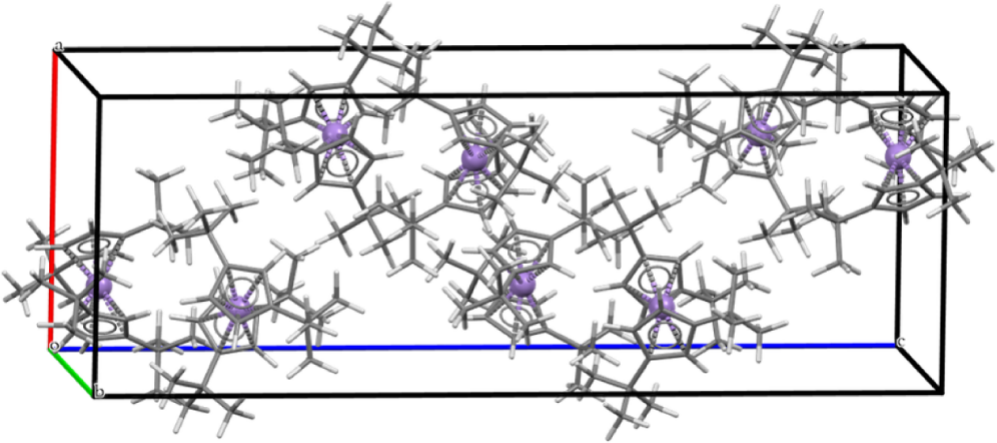

Spin energetics is one of the biggest challenges associated with energy calculations for electronic structure methods. The energy differences of the spin states in spin-crossover compounds are very small, making them one of the most difficult systems to calculate. Few methods provide accurate results for calculating these energy differences. In addition, studies have usually focused on calculating energetics of single molecules, while spin-crossover properties are usually experimentally studied in the solid phase. In this paper, we have used periodic boundary conditions employing methods based on density functional theory to calculate the high- and low-spin energy differences for a test case of 20 extended systems. Compounds with different metals and ligands have been selected, and the results indicate that a semiquantitative description of the energy differences can be obtained with the combination of geometry optimization using the PBE functional including many-body dispersion approach and the use of meta-GGA functionals, such as r^2^SCAN but especially KTBM24, for the energy calculation. Other hybrid functionals, such as TPSSh, give generally good results, but the calculation of the exact exchange with periodic boundary conditions involves a huge increase in computer time and computational resources. It makes the proposed nonhybrid functional approach (KTBM24//PBE+MB) a great advantage for the study of periodic systems.

## Abstract 2

A set of 20 compounds presenting spin transition with different metals and ligands has been used to benchmark periodic DFT methods. A new nonhybrid trained meta-GGA functional KTBM24 gives better results than the functionals recommended for this type of systems including hybrid functionals. It is especially suitable for periodic systems as it does not have to calculate the exact exchange and allows the calculation of unit cell systems with numerous molecules.

## 1 Introduction

Spin-crossover systems present extraordinary characteristics that make them interesting both from an experimental point of view, where their switching capabilities find applications in electronic devices, and from a theoretical perspective, due to the computational challenges associated with the precise calculation of the energy differences between spin states.^1−3^ From an experimental point of view, the characterization of thousands of compounds with this property has mainly involved magnetic measurements to determine the transition temperature (*T*_1/2_) between low- and high-spin states.^4^ In many cases, there is also the hysteresis of the magnetization with temperature, which gives them additional potential applications.^3,5^ This behavior was normally assigned to the difference in the cooperative interactions between neighboring molecules in the high- and low-spin states, although recently it has been observed hysteresis behavior in a single-molecule complex.^6^

From the theoretical point of view, these systems still have many open challenges.^7^ Although magnetic properties are often determined on solid-state powder samples, many computational studies focus only on molecular calculations mainly due to the computational resources needed.^8,9^ Second, the calculation of *T*_1/2_ requires taking into account entropic contributions, which leads to the necessity of the calculation of vibrational energies.^10,11^ This is particularly demanding due to the sensitivity of the entropic contributions to small errors in the vibrational energies.^12^ Furthermore, in periodic boundary condition (PBC) approximations, equivalent phonon calculations pose challenges, given the absence of analytical derivatives.^8^ Third, despite that numerous theoretical approaches have been explored to calculate the electronic energy difference between low- and high-spin states, surprisingly, high-precision post-Hartree–Fock methods based on complete active space self-consistent field (CASSCF), including dynamical correlation such as CASPT2 or NEVPT2, do not consistently outperform density functional theory (DFT) approaches.^13^ This is mainly because the Hartree–Fock starting function produces a high overstabilization of the high-spin state;^14^ thus, nonconventional methods, as combinations of CASPT2 with coupled cluster and MRCI approaches, must be considered.^15−19^ In general spin-transition systems pose a demanding test of accuracy for the computational approaches because the low-spin state must be about 1–10 kcal/mol more stable than the high-spin state at 0 K, and the compensation with entropic contributions allows the system to exhibit spin-crossover behavior at higher temperatures.^20,21^

The quantitative calculation of transition temperatures faces several drawbacks, prompting the DFT method as a potential solution. DFT allows for both molecular and periodic systems calculations,^9,22^ offering analytic gradient and frequency calculations in molecular systems and numerical approaches in periodic structures.^8,23,24^ Despite several implementations of some post-Hartree–Fock methods with periodic boundary conditions, such approaches are difficult to consider because they face limitations in handling large systems and performing phonon calculations.^25,26^ Furthermore, DFT provides relatively accurate energy differences between low- and high-spin states even better than such ab initio methods.^8,27−29^ In recent works,^8,9,27,28^ a benchmark set of 20 spin-crossover systems was established, spanning all first-row transition metals showing such property and each of them exhibiting a well-defined abrupt low-high-spin transition.

Among the plethora of exchange correlation functionals tested since the first DFT studies of molecular spin-crossover systems,^20,30−35^ the hybrid meta-GGA TPSSh functional^36,37^ consistently described the ground state for the entire set, demonstrating relatively small high- and low-spin energy differences.^27^ Hybrid functionals pose a major problem when dealing with periodic systems, as the exchange term requires a lot of computational resources both in terms of time and memory.^26,38^ In this sense, the meta-GGA functionals of the SCAN family have recently emerged as a good alternative,^39−42^ since the results are similar to those obtained with the TPSSh functional but without including the exchange term. Within this family, the best results are obtained with the r^2^SCAN functional.^28,43,44^ However, we have tried the performance of a family of functionals recently developed by Kóvacs et al. for molecular spin-crossover systems.^45^ These functionals are a new series of meta-GGA-type training functionals specially dedicated to reproduce cell parameters, cohesive energies and bandgaps.^46^ These authors have proposed 25 combinations of three coefficients that control the reliability with which each of the three magnitudes previously mentioned can be reproduced. It generates 25 expressions for the exchange part of the functional (KTBM0-KTBM24, Kovács-Tran-Blaha-Madsen), while the correlation contribution is included with the SCAN functional. One of these functionals, KTBM24,^46^ provides very good values for spin energy differences of molecular spin-crossover systems, even better than TPSSh or r^2^SCAN.^45^

This work aims to analyze the accuracy of meta-GGA functionals, especially the r^2^SCAN and KTBM24 functionals, to reproduce the energy differences between low- and high-spin states in a benchmark set of 20 periodic systems. Previously for molecular calculations, a set of 20 systems was already proposed, but in the case of the periodic structures, three of them have been substituted due to a lack of crystal structures or in one case for a huge unit cell with a bad structural resolution.^8,27−29^ The availability of accurate calculation methods is crucial for spin energetics of metal complexes in the field of spin-crossover systems but also vital for the correct description of the local spins in single-molecule magnets,^47,48^ bioinorganic systems,^49^ catalysis involving metal spin,^50^ and a long etcetera of cases.

## 2 Computational Details

DFT calculations were performed with the all-electron FHI-aims computer code using numerical local orbital basis sets for either molecular or periodic systems.^51^ Molecular calculations were performed with different types of GGA and meta-GGA functionals. Different options have been employed and analyzed, such as optimizing the structure at the GGA level and determining the energies at the GGA or meta-GGA level. Thus, the geometry optimizations for the periodic structures were done using the PBE exchange-correlation functional^52^ including dispersion effects through a many-body approach (PBE+MB approach)^53^ and using such optimized structures for the energy calculation with the meta-GGA r^2^SCAN or KTBM24 functionals (r^2^SCAN//PBE+MB and KTBM24//PBE+MB methods). Different numerical basis sets and grids have been tested with molecular calculations to check their influence (Table S1). Scalar relativistic effects were included using the scaled zeroth order regular approximation (ZORA) approach.^54^ The number of k-points and the split grids for the reciprocal space employed in the periodic calculations were tested and are collected in Table S2. The KTBM24 despite including the correlation contribution using the SCAN functional does not show the problem to require a very large grid to converge the results indicating that this drawback is mainly related to the exchange part of the SCAN functional, as already noticed.^41,42^

To make a comparison between experimental data and theoretical results for all the systems, the transition temperature was used, which is the experimental available data (see Table S3) for all the systems studied. At this temperature, the free energy of the system at high-spin and low-spin states has the same value because the entropy compensates for the more stable low spin at low temperature (see eq 2). Thus, we can calculate the thermal correction (*E*^therm^ in eq 1, which is the value to be added to the energy to obtain the free energy) of each of the two spin states. The difference between them must be equal to the expected high- and low-spin energy splitting that would reproduce the experimental transition temperature (eq 3). Hence, such energy can be employed as a semiquantitative experimental value to facilitate the comparison with the calculated values.
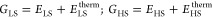
1

At the transition temperature *T*_1/2_
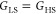
2and consequently,
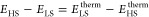
3

The calculations to obtain such estimation of the spin state energy difference were performed by calculating the thermal energy correction at the transition temperature for the two states of each system using the Gaussian16 code^55^ with the hybrid B3LYP functional^56^ using the 6-311G basis set^57^ and the D3 approach^58^ to include the dispersion contributions (Table S3). The B3LYP results were employed because it was considered the best approach to reproduce the vibrational frequencies (scaling parameter closest to one); being this term the main contribution to the thermal energy differences through the zero-point correction and entropic terms.^59^ Therefore, the difference of the thermal correction for the high- and low-spin states obtained with B3LYP was included in the last column of Table 1 to compare with the calculated energy differences between low- and high-spin states.

**Table 1:** High-and low-spin state energy differences (in kcal/mol) per molecule using periodic structures (positive values indicate a low-spin ground state)^a^^b^^c^^d^

crystal system	ref.	PBE+MB	r^2^SCAN// PBE+MB	r^2^SCAN+MB// PBE+MB	KTBM24// PBE+MB	*T*_1/2_
**s1** [Cr^II^(L_s1_)_2_I_2_]	(65)	6.4 (15.1)	0.45 (5.7)	4.5 (8.8)	0.1 (5.8)	1.7
**s2new** [Mn^III^(L_s2new_)][AsF_6_]	(60)	15.0 (12.0)	6.6 (4.0)	9.6 (4.4)	7.1 (4.6)	2.5
**s3** [Mn^III^(L_s3_)][BF_4_]	(66)	11.2 (13.4)	6.8 (5.5)	10.7 (8.8)	6.9 (5.7)	2.7
**s4** [Mn^III^(L_s4_)][PF_6_]	(67)	12.0 (12.1)	6.1 (3.1)	9.4 (6.2)	6.7 (3.5)	2.3
**s5new** [Mn^III^(L_s5new_)_2_]Cl·3H_2_O	(61)	19.2 (10.9)	7.5 (1.6)	10.9 (14.8)	3.9 (2.3)	2.9
**s6** [Mn^II^(L_s6_)_2_]	(68)	25.9 (29.0)	8.2 (10.4)	10.5 (13.2)	8.8 (11.4)	6.4
**s7** [Mn^II^(L_s7_)_2_]	(68)	25.3 (29.2)	10.7 (10.3)	14.4 (14.0)	12.0 (11.6)	9.2
**s8new** [Fe^III^(L_s8new_)_2_][ClO_4_]·H_2_O	(62)	25.0 (28.3)	8.8 (12.2)	9.4 (7.2)	6.8 (9.4)	2.7
**s9** [Fe^III^(L_s9a_)(L_s9b_)_2_][ClO_4_]	(69)	21.9 (28.8)	10.7 (12.6)	16.4 (17.0)	10.9 (13.8)	4.0
**s10** [Fe^III^(L_s10_)_2_][PF_6_]	(70)	21.8 (23.8)	9.4 (8.6)	12.7 (13.0)	11.1 (9.5)	3.1
**s11** [Fe^II^(L_s11_)_2_(NCS)_2_]	(71)	23.1 (23.1)	9.8 (7.4)	12.6 (10.2)	6.4 (3.6)	4.3
**s12** [Fe^II^(L_s12_)_4_(NCS)_2_]	(72)	21.6 (24.3)	11.4 (9.4)	17.9 (15.4)	8.9 (6.2)	5.4
**s13** [Fe^II^(L_s13_)_2_][BF_4_]_2_	(73,74)	29.7 (30.0)	16.5 (14.0)	20.7 (18.5)	9.5 (8.2)	5.3
**s14** [Fe^II^(L_s14a_)(L_s14b_)]	(75)	21.2 (28.3)	11.3 (12.6)	17.4 (18.0)	8.1 (9.3)	4.0
**s15** [Fe^II^(L_s15_)_2_(NCS)_2_]	(76)	27.8 (20.1)	12.1 (5.8)	16.3 (6.3)	9.6 (2.8)	1.7
**s16** [Co^II^(L_s16_)_2_]I_2_·4H_2_O	(77)	24.3 (20.0)	11.2 (9.6)	13.5 (12.0)	4.6 (2.3)	2.0
**s17** [Co^II^(L_s17_)(Py)_2_]	(78)	15.4 (15.2)	9.3 (7.8)	11.2 (8.9)	3.2 (2.8)	1.2
**s18** [Co^II^(L_s18_)_2_][ClO_4_]_2_·H_2_O	(79),^80^	20.5 (20.8)	9.5 (8.7)	11.4 (11.0)	2.4 (1.3)	1.6
**s19** [Co^II^(L_s19_)_2_]	(81)	19.4 (25.0)	11.7 (15.1)	13.6 (18.1)	4.2 (7.1)	1.5
**s20** [Co^II^(L_s20_)_2_][BF_4_]_2_·H_2_O·EtOH	(82)	21.5 (20.0)	9.3 (10.3)	10.5 (12.7)	2.3 (5.7)	1.7

a If two functionals are indicated, the first one was employed in the energy calculations and the second one in the optimization of the structure.

b MB indicates the inclusion of many-body dispersion in the geometry optimization.

c Values in parenthesis correspond to the energy difference for equivalent discrete molecular systems with the same theoretical method.

d Last column corresponds to the energy difference between high- and low-spin states obtained from eq 3^3^ for the discrete molecular systems and considering the thermal energy corrections to reproduce the experimental T 1/2. Ligand names are in Figure 1 caption.

## 3 Results and Discussion

### 3.1 Molecular Systems

As mentioned above, a set of 20 spin-crossover systems has been used to check the accuracy of the theoretical approaches by performing calculations with the isolated molecules. For the theoretical study using periodic calculations to be presented in the next section, three systems, s2, s5, and s8 (see Figure 1), have been replaced by three new systems (s2new,^60^ s5new,^61^ and s8new)^62^ as no crystal structural data are available for s5 and s8, while s2 has a huge unit cell with 16 molecules with relatively high flexibility that makes very difficult the geometry optimization. In this system,^63,64^ in the X-ray single-crystal structure, there are quite different distances between the metal and the atoms of the first coordination sphere between the 16 molecules showing a high dispersion in the structural parameters.

**Figure 1:**
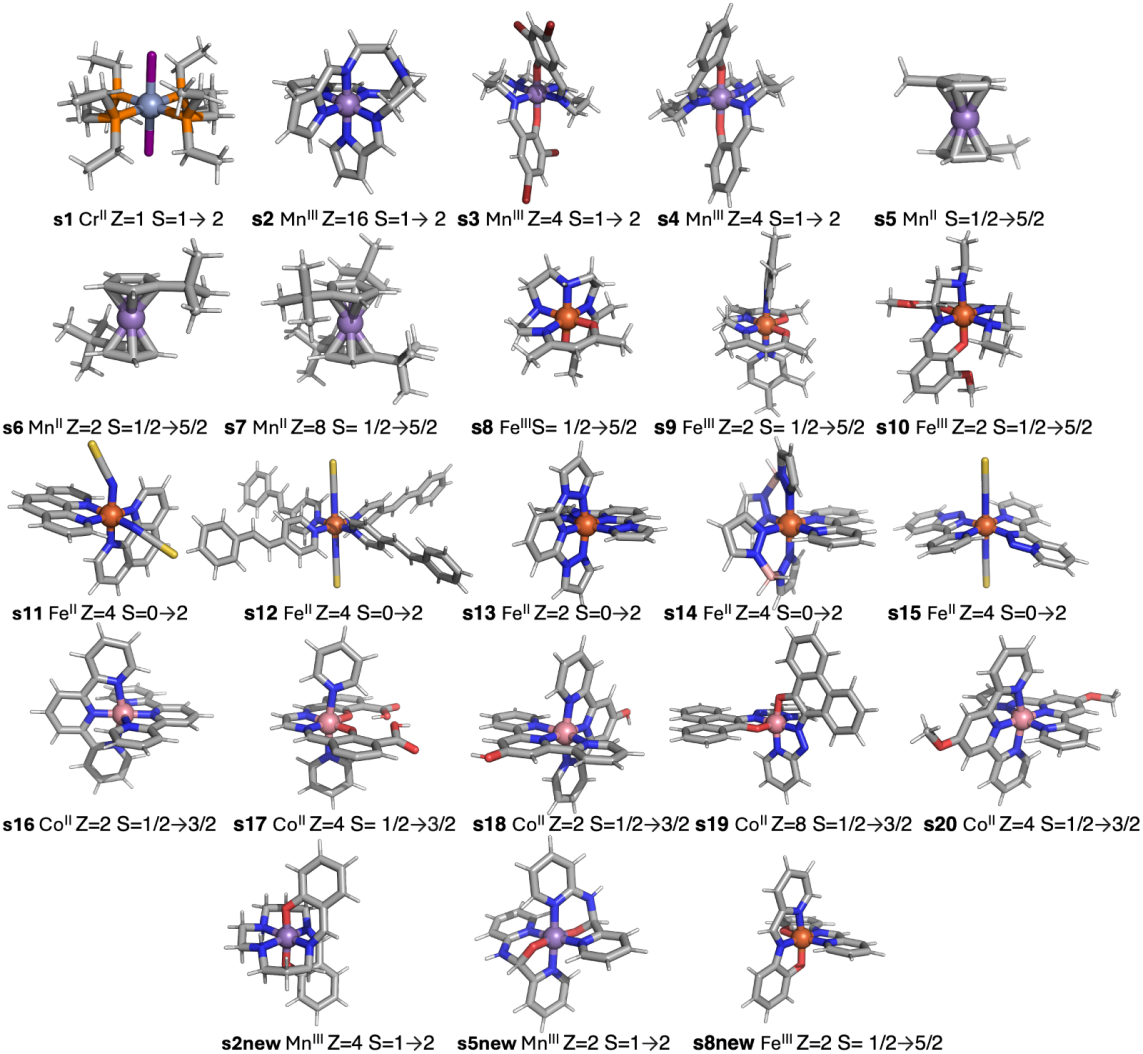
Representation of the spin-crossover systems employed as benchmark set, indicating the label, the number of molecules in the unit cell, and the spin of the low- and high-spin states. Ligands for the 23 systems: L_s1_ = 1,2-bis(diethylphosphino)ethane-P,P’, L_s2_ = tris(2-((pyrrol-2-yl)methyleneamino)ethyl)amine, L_s3_ = 2,2′-(2,6,9,13-tetraazatetradeca-1,13-diene-1,14-diyl)bis(4,6-dibromophenolato), L_s4_ = 2,2′-(2,6,9,13-tetra-azatetradeca-1,13-diene-1,14-diyl)bis(phenolato), L_s5_ = η^5^-methylcyclopentadiene, L_s6_ = η^5^-*tert*-butylcyclopentadienyl, L_s7_ = η^5^-1,3-*tert*-butylcyclopentadienyl, L_s8_ = *N^1^,N^4^*-bis(acetylacetonato)triethylenetetramine, L_s9a_ = ethylenebis(acetylacetoneiminato), L_s9b_ = 3,4-dimethylpyridyl, L_s10_= 2-(((2-(ethylamino)ethyl)imino)methyl)-6-methoxyphenolato-N,*N*′,O, L_s11_ = bis(1,10-phenanthroline-N,N’), L_s12_ = 4-styrylpyridine, L_s13_ = (2,6-dipyrazol-1-yl)pyridine), L_s14a_ = dihydrogen bis(pyrazol-1-yl)borate, L_s14b_ = 2,2′-bipyridyl, L_s15_ = 3-(2-pyridyl)(1,2,3)triazolo(1,5-a)pyridine, L_s16_= 2,2’:6′,2’’-terpyridine, L_s17_ = 3-formylsalicylic acid-ethylendiamine, L_s18_ = 2,2’:6′,2’’-terpyridin-4′-ol), L_s19_ = 10-((pyridin-2-yl)diazenyl)phenanthren-9-olato, L_s20_ = 4′-methoxy-2,2’:6′,2’’-terpyridine, L_s2new_ = Schiff-base (sal-N-1,5,8,12)^2–^, L_s5new_ = (2-pyridiyl)(2-pyridylamino)methoxy, L_s8new_ = deprotonated bis[2-hydroxyphenyl-(2-pyridyl)-methaneimine.

One of the usual criteria for assessing the reliability of the calculation methods is the number of cases with an energy difference outside the range 0–10 kcal/mol (the low-spin state being more stable the low-spin state). This is the energy that can be compensated for by increasing the temperature and stabilizing the high-spin state. However, as we can see from the results using eq 3, last column in Table 1, in most cases, the values of the high- and low-spin energy difference are below 4 kcal/mol, and only in a few isolated cases with relatively high transition temperatures does the energy difference approach 10 kcal/mol. In this case, the use of meta-GGA functionals is fundamental (see Table 1, values between parentheses) as it has been previously studied.^28,43,44^

**Figure 2:**
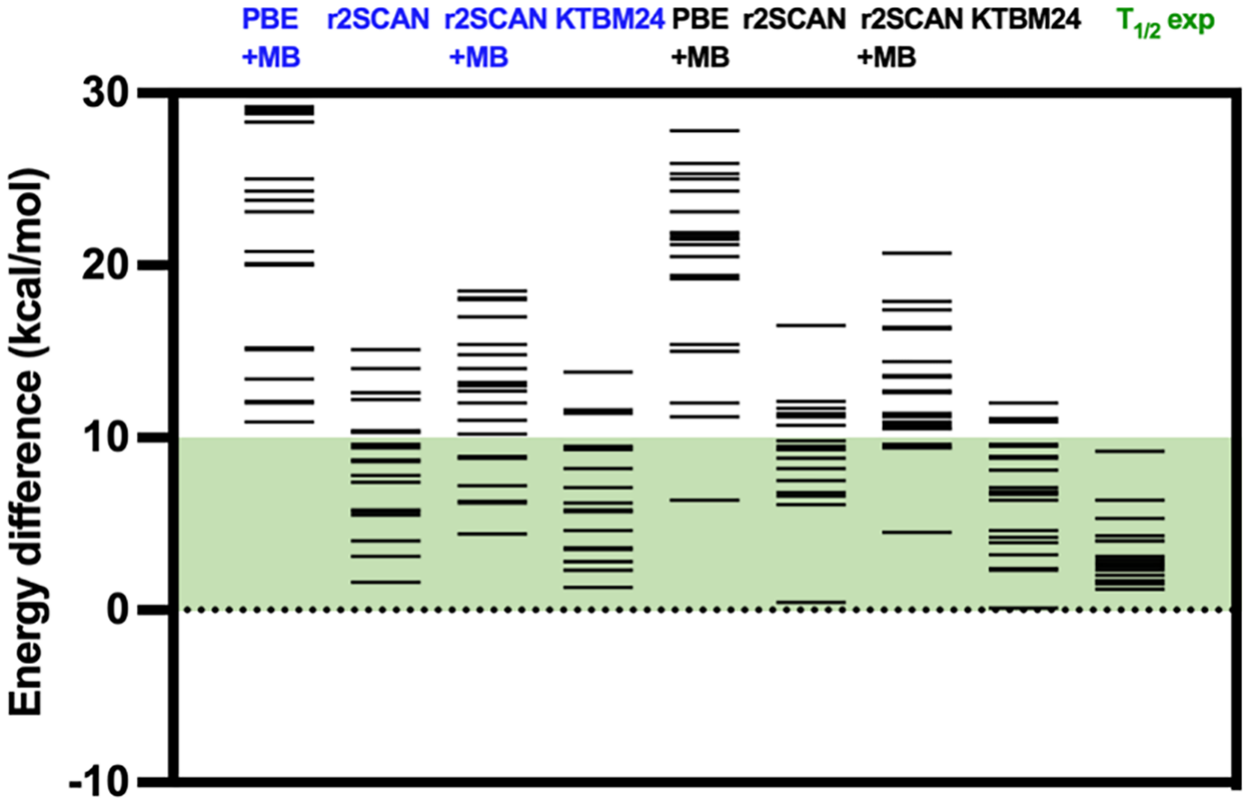
High- and low-spin energy differences for the 20 test cases employed in Table 1 with different exchange-correlation functionals for molecules (blue) and for periodic systems (black). The results for the PBE+MB, r^2^SCAN and KTBM24 functionals were obtained by optimizing the geometry with PBE+MB and calculating a single-point energy with the meta-GGA functional. The values under the *T*_1/2_ label correspond to those energy differences in reproducing the experimental transition temperatures. Green energy range of 10 kcal/mol was usually considered as an energy difference that can be compensated by the entropy in spin-crossover compounds.

As mentioned in the Introduction section, r^2^SCAN provide an accuracy close to the hybrid TPSSh functional but without the calculation of exact-type exchange.^24,25^ The inclusion of the dispersion term in the meta-GGA functional causes an overstabilization of the low spin state resulting in worse results (see Table 1 and Figure 2).^28,40^ The stability of the results was verified with the r^2^SCAN//PBE+MB and KTBM24//PBE+MB methodologies by increasing the grid size and the quality of the base (from *tight* to *very tight*). These improvements provide negligible variations in the calculated energy difference (Table S1). The comparison between the two meta-GGA functionals, r^2^SCAN and KTBM24, reveals that for **s1–s10** molecular systems, the results are relatively similar. However, for Fe^II^ and Co^II^ molecules (**s11–s20**), KTBM24 gives results much closer to the estimate from the transition temperatures while r^2^SCAN slightly overestimates the stability of the low-spin state.

### 3.2 Extended Systems

The set case has been studied, including periodic boundary conditions and using the experimental data of elementary cells and atomic positions determined by X-ray diffraction as starting structures. In this type of calculations, the meta-GGA functional (see Table 1) constitutes an excellent compromise compared to the TPSSh functional, since the calculation of the exact exchange term requires a lot of computational resources. The conclusions drawn from molecular calculations hold when extended systems. Although computations with the meta-GGA functionals require much fewer computational resources than hybrid functionals, performing geometry optimizations with it still requires considerable computational time. In this sense, the combination with a GGA functional that provides good results in geometry optimizations at a low computational cost is the best option. Furthermore, we have seen at the level of molecular calculations that the differences in energy and optimized geometrical parameters between the PBE+MB and r^2^SCAN methods are very small (see SI). Thus, the combination of optimizing at the PBE plus dispersion level and calculating the energy with the meta-GGA functional provides excellent results for the high- and low-spin energy differences (Table 1). Although the PBE functional gives results with a high overestimation of the low spin stability, it is noteworthy that the relative values follow a qualitatively similar trend to those obtained with the meta-GGA functionals. Comparison of the results for periodic systems with the two meta-GGA functionals, r2SCAN and KTBM24, shows a similar trend as with the molecular models. The values for the Fe^III^ and Mn^III^ systems are relatively similar, although slightly lower with KTBM24 and closer to the estimated energy difference with the experimental transition temperature. But with the Fe^II^ and Co^II^ systems, the difference between the two functionals is larger and the values obtained with KTBM24 are closer to the experimentally expected values. A comparison of these methodologies between molecular and periodic calculations does not show a clear trend in the variation of the high- and low-spin energy difference with packing. The energy difference diminishes with the packing in practically half of the cases depending on the DFT method. In the rest, it increases when passing from the molecule to the solid (Table 1 and Figure 2).

Analysis of the structures indicates that in most cases, especially in the smaller low-spin structures, the optimized geometries for the molecules are very similar to those obtained by packing the molecules (see the metal–ligand distances in Supporting Information 2). However, there is one exception within the case set which is the Mn^II^ metallocenes. In these cases, it is known that the isolated molecules either using gas-phase electron diffraction or calculations of the isolated molecules lead to an eclipsed arrangement of the cyclopentadiene ligands. But in the optimized structure of the solid structures or experimentally using single-crystal X-ray diffraction, the cyclopentadiene ligands are arranged staggered. In the high-spin state of the molecular manganocene calculations, weak metal–ligand bonding leads to very distorted structures and also relative arrangements of the substituents of the cyclopentadiene ligands that are difficult to avoid if the molecule is not packed in the crystal.

When comparing the unit cell volumes optimized with PBE+MB together with the available experimental values (Table S2), it can be seen that the volume increase at spin transitions is typically below 5% in the calculated values. However, experimentally, the variations obtained are larger. This indicates that the calculated values for the cells in high-spin states are too small. This may be mainly because the geometry optimization at the DFT level is performed in the high-spin state as if it were at 0 K temperature. It must also be considered the thermal effects on the structures that produce an increase in volume. For instance, in the **s13** system^74,83^ which transitions very abruptly at 256 K, the volume change with the low-spin structure between 30 and 240 K is of the order of 3%, while the change between 240 and 300 K with the transition is approximately the same. This indicates that thermal effects over a wide temperature range can cause a structural change equivalent to a spin transition. All of this affects the comparison of experimental data with the results of optimizations with theoretical methods.

## 4 Concluding Remarks

The main objective has been to verify the reliability of methods based on density functional theory to calculate high- and low-spin energy differences in spin-crossover systems employing periodic structures. The calculation of these systems involves important challenges since in many cases, the unit cells of these systems contain a large number of molecules. This makes post-Hartree–Fock methods unadvisable, as essentially they can only be used with discrete molecules and they also have a problem of overestimating the stability of high-spin states. It converts DFT methods as the most plausible option to calculate systems with many atoms and periodic properties. Previous studies have shown that the two functionals that present high accuracy in the calculation of energy differences of spin-crossover compounds are the hybrid meta-GGA TPSSh functional and the meta-GGA r^2^SCAN. The TPSSh functional is recommended for molecular systems, but the calculation of the exact exchange in periodic systems is a major problem both in the accuracy of the calculation and in the high computational resources even to calculate only the energy. We show that the recently developed KTBM24 functional improves both the results of the r^2^SCAN functional at the level of periodic calculations and even those obtained with the hybrid TPSSh at the molecular level.^27^ Hence, an important alternative to have a good estimation of the spin energetics is to use a GGA functional such as PBE including dispersion terms to optimize the geometry and then calculate the energy with the KTBM24 functional, as we propose here. Additionally, we have used a new criterion calculating the energy difference between the two states that reproduces the experimental transition temperature calculated as the difference in thermal energy corrections.

## Supporting Information Available

High- and low-spin states energy differences using discrete molecular models with different grids and basis sets (Table S1); k-points in the periodic calculations, and DFT optimized and experimental unit cell volumes (Table S2); the experimental *T*_1/2_ values and the energy estimated with this critical temperature (Table S3) (PDF)
The calculated and experimental bond distances (XLSX)
*xyz* and *cif* files with the optimized structures (ZIP)

## References

[ref1] Gütlich P.; Goodwin H. A. Spin Crossover—An Overall Perspective. In Spin Crossover in Transition Metal Compounds, Gütlich P.; Goodwin H. A. Eds.; Springer: Berlin Heidelberg: 2004, pp. 1 47.

[ref2] Halcrow M. A. Structure: Function Relationships in Molecular Spin-Crossover Materials. In Spin-Crossover Materials; Wiley, 2013. 10.1039/c1cs15046d 21483934

[ref3] Senthil Kumar K.; Ruben M. Emerging trends in spin crossover (SCO) based functional materials and devices. Coord. Chem. Rev. 2017, 346, 176–205. 10.1016/j.ccr.2017.03.024.

[ref4] Halcrow M. A. Structure: Function relationships in molecular spin-crossover complexes. Chem. Soc. Rev. 2011, 40, 4119–4142. 10.1039/c1cs15046d. 21483934

[ref5] Bousseksou A.; Molnár G.; Salmon L.; Nicolazzi W. Molecular spin crossover phenomenon: recent achievements and prospects. Chem. Soc. Rev. 2011, 40, 3313–3335. 10.1039/c1cs15042a. 21544283

[ref6] Moneo-Corcuera A.; Nieto-Castro D.; Cirera J.; Gómez V.; Sanjosé-Orduna J.; Casadevall C.; Molnár G.; Bousseksou A.; Parella T.; Martínez-Agudo J. M.; et al. Molecular memory near room temperature in an iron polyanionic complex. Chem 2023, 9, 377–393. 10.1016/j.chempr.2022.09.025.

[ref7] Ruiz E. Charge transport properties of spin crossover systems. Phys. Chem. Chem. Phys. 2014, 16, 14–22. 10.1039/C3CP54028F. 24217339

[ref8] Cirera J.; Ruiz E. Assessment of the SCAN Functional for Spin-State Energies in Spin-Crossover Systems. J. Phys. Chem. A 2020, 124, 5053–5058. 10.1021/acs.jpca.0c03758. 32449616

[ref9] Albavera-Mata A.; Hennig R. G.; Trickey S. B. Transition Temperature for Spin-Crossover Materials with the Mean Value Ensemble Hubbard-U Correction. J. Phys. Chem. A 2023, 127, 7646–7654. 10.1021/acs.jpca.3c03520. 37669434

[ref10] Cirera J.; Paesani F. Theoretical Prediction of Spin-Crossover Temperatures in Ligand-Driven Light-Induced Spin Change Systems. Inorg. Chem. 2012, 51, 8194–8201. 10.1021/ic300750c. 22817277

[ref11] Römer A.; Hasecke L.; Blöchl P.; Mata R. A. A Review of Density Functional Models for the Description of Fe(II) Spin-Crossover Complexes. Molecules 2020, 25, 5176 10.3390/molecules25215176. 33172067 PMC7664392

[ref12] Cirera J.; Ruiz E. Computational Modeling of Transition Temperatures in Spin-Crossover Systems. Comments Inorg. Chem. 2019, 39, 216–241. 10.1080/02603594.2019.1608967.

[ref13] Radon M. Benchmarking quantum chemistry methods for spin-state energetics of iron complexes against quantitative experimental data. Phys. Chem. Chem. Phys. 2019, 21, 4854–4870. 10.1039/C9CP00105K. 30778468

[ref14] Song S.; Kim M. C.; Sim E.; Benali A.; Heinonen O.; Burke K. Benchmarks and Reliable DFT Results for Spin Gaps of Small Ligand Fe(II) Complexes. J. Chem. Theory Comput. 2018, 14, 2304–2311. 10.1021/acs.jctc.7b01196. 29614856

[ref15] Phung Q. M.; Feldt M.; Harvey J. N.; Pierloot K. Toward Highly Accurate Spin State Energetics in First-Row Transition Metal Complexes: A Combined CASPT2/CC Approach. J. Chem. Theory Comput. 2018, 14, 2446–2455. 10.1021/acs.jctc.8b00057. 29614218

[ref16] Pierloot K.; Phung Q. M.; Domingo A. Spin State Energetics in First-Row Transition Metal Complexes: Contribution of (3s3p) Correlation and Its Description by Second-Order Perturbation Theory. J. Chem. Theory Comput. 2017, 13, 537–553. 10.1021/acs.jctc.6b01005. 28005368

[ref17] Mariano L. A.; Vlaisavljevich B.; Poloni R. Biased Spin-State Energetics of Fe(II) Molecular Complexes within Density-Functional Theory and the Linear-Response Hubbard U Correction. J. Chem. Theory Comput. 2020, 16, 6755–6762. 10.1021/acs.jctc.0c00628. 33108722

[ref18] Mariano L. A.; Vlaisavljevich B.; Poloni R. Improved Spin-State Energy Differences of Fe(II) Molecular and Crystalline Complexes via the Hubbard U-Corrected Density. J. Chem. Theory Comput. 2021, 17, 2807–2816. 10.1021/acs.jctc.1c00034. 33831303

[ref19] Reimann M.; Kaupp M. Spin-State Splittings in 3d Transition-Metal Complexes Revisited: Benchmarking Approximate Methods for Adiabatic Spin-State Energy Differences in Fe(II) Complexes. J. Chem. Theory Comput. 2022, 18, 7442–7456. 10.1021/acs.jctc.2c00924. 36417564

[ref20] Brehm G.; Reiher M.; Schneider S. Estimation of the vibrational contribution to the entropy change associated with the low- to high-spin transition in Fe(phen)_2_(NCS)_2_ complexes: Results obtained by IR and Raman spectroscopy and DFT calculations. J. Phys. Chem. A 2002, 106, 12024–12034. 10.1021/jp026586o.

[ref21] Ye S.; Neese F. Accurate Modeling of Spin-State Energetics in Spin-Crossover Systems with Modern Density Functional Theory. Inorg. Chem. 2010, 49, 772–774. 10.1021/ic902365a. 20050628

[ref22] Mejía-Rodríguez D.; Albavera-Mata A.; Fonseca E.; Chen D.-T.; Cheng H. P.; Hennig R. G.; Trickey S. B. Barriers to predictive high-throughput screening for spin-crossover. Comput. Mater. Sci. 2022, 206, 111161 10.1016/j.commatsci.2021.111161.

[ref23] Siig O. S.; Kepp K. P. Iron(II) and Iron(III) Spin Crossover: Toward an Optimal Density Functional. J. Phys. Chem. A 2018, 122, 4208–4217. 10.1021/acs.jpca.8b02027. 29630380

[ref24] Kepp K. P. Consistent descriptions of metal-ligand bonds and spin-crossover in inorganic chemistry. Coord. Chem. Rev. 2013, 257, 196–209. 10.1016/j.ccr.2012.04.020.

[ref25] McClain J.; Sun Q.; Chan G. K.-L.; Berkelbach T. C. Gaussian-Based Coupled-Cluster Theory for the Ground-State and Band Structure of Solids. J. Chem. Theory Comput. 2017, 13, 1209–1218. 10.1021/acs.jctc.7b00049. 28218843

[ref26] Ren X.; Rinke P.; Blum V.; Wieferink J.; Tkatchenko A.; Sanfilippo A.; Reuter K.; Scheffler M. Resolution-of-identity approach to Hartree–Fock, hybrid density functionals, RPA, MP2 and GW with numeric atom-centered orbital basis functions. New J. Phys. 2012, 14, 053020 10.1088/1367-2630/14/5/053020.

[ref27] Cirera J.; Via-Nadal M.; Ruiz E. Benchmarking Density Functional Methods for Calculation of State Energies of First Row Spin-Crossover Molecules. Inorg. Chem. 2018, 57, 14097–14105. 10.1021/acs.inorgchem.8b01821. 30383364

[ref28] Mejía-Rodríguez D.; Trickey S. B. Spin-Crossover from a Well-Behaved, Low-Cost meta-GGA Density Functional. J. Phys. Chem. A 2020, 124, 9889–9894. 10.1021/acs.jpca.0c08883. 33175542

[ref29] Mejia-Rodriguez D.; Trickey S. B. Deorbitalization strategies for meta-generalized-gradient-approximation exchange-correlation functionals. Phys. Rev. A 2017, 96, 052512 10.1103/PhysRevA.96.052512.

[ref30] Fouqueau A.; Casida M. E.; Daku L. M. L.; Hauser A.; Neese F. Comparison of density functionals for energy and structural differences between the high- [^5^T_2g_: (t_2g_)^4^(e_g_)^2^] and low- [^1^A_1g_: (t_2g_)^6^(e_g_)] spin states of iron(II) coordination compounds. II. More functionals and the hexaminoferrous cation, [Fe(NH_3_)_6_]^2+^. J. Chem. Phys. 2005, 122 (4), 044110 10.1063/1.1839854. 15740238

[ref31] Fouqueau A.; Mer S.; Casida M. E.; Hauser A.; Mineva T.; Neese F. Comparison of density functionals for energy and structural differences between the high- [^5^T_2g_: (t_2g_)^4^(e_g_)^2^] and low- [^1^A_1g_: (t_2g_)^6^(e_g_)] spin states of the hexaquoferrous cation [Fe(H_2_O)_6_]^2+^. J. Chem. Phys. 2004, 120, 9473–9486. 10.1063/1.1710046. 15267959

[ref32] Swart M. Accurate Spin-State Energies for Iron Complexes. J. Chem. Theory Comput. 2008, 4, 2057–2066. 10.1021/ct800277a. 26620478

[ref33] Swart M.; Gruden M. Spinning around in Transition-Metal Chemistry. Acc. Chem. Res. 2016, 49, 2690–2697. 10.1021/acs.accounts.6b00271. 27993008

[ref34] Swart M.; Güell M.; Solà M. A multi-scale approach to spin crossover in Fe(ii) compounds. Phys. Chem. Chem. Phys. 2011, 13, 10449–10456. 10.1039/c1cp20646j. 21505661

[ref35] Kepp K. P. Theoretical Study of Spin Crossover in 30 Iron Complexes. Inorg. Chem. 2016, 55, 2717–2727. 10.1021/acs.inorgchem.5b02371. 26913489

[ref36] Tao J. M.; Perdew J. P.; Staroverov V. N.; Scuseria G. E. Climbing the Density Functional Ladder: Nonempirical Meta–Generalized Gradient Approximation Designed for Molecules and Solids. Phys. Rev. Lett. 2003, 91 (14), 146401 10.1103/PhysRevLett.91.146401. 14611541

[ref37] Staroverov V. N.; Scuseria G. E.; Tao J.; Perdew J. P. Comparative assessment of a new nonempirical density functional: Molecules and hydrogen-bonded complexes. J. Chem. Phys. 2003, 119, 12129–12137. 10.1063/1.1626543.

[ref38] Levchenko S. V.; Ren X.; Wieferink J.; Johanni R.; Rinke P.; Blum V.; Scheffler M. Hybrid functionals for large periodic systems in an all-electron, numeric atom-centered basis framework. Comput. Phys. Commun. 2015, 192, 60–69. 10.1016/j.cpc.2015.02.021.

[ref39] Sun J.; Ruzsinszky A.; Perdew J. P. Strongly Constrained and Appropriately Normed Semilocal Density Functional. Phys. Rev. Lett. 2015, 115, 036402 10.1103/PhysRevLett.115.036402. 26230809

[ref40] Kothakonda M.; Kaplan A. D.; Isaacs E. B.; Bartel C. J.; Furness J. W.; Ning J.; Wolverton C.; Perdew J. P.; Sun J. Testing the r^2^SCAN Density Functional for the Thermodynamic Stability of Solids with and without a van der Waals Correction. ACS Mater. Au. 2023, 3, 102–111. 10.1021/acsmaterialsau.2c00059. 38089726 PMC9999476

[ref41] Furness J. W.; Kaplan A. D.; Ning J.; Perdew J. P.; Sun J. Construction of meta-GGA functionals through restoration of exact constraint adherence to regularized SCAN functionals. J. Chem. Phys. 2022, 156, 034109 10.1063/5.0073623. 35065548

[ref42] Furness J. W.; Kaplan A. D.; Ning J.; Perdew J. P.; Sun J. Accurate and Numerically Efficient r^2^SCAN Meta-Generalized Gradient Approximation. J. Phys. Chem. Lett. 2020, 11, 8208–8215. 10.1021/acs.jpclett.0c02405. 32876454

[ref43] Wu S.-G.; Wang L.-F.; Ruan Z.-Y.; Du S.-N.; Gómez-Coca S.; Ni Z.-P.; Ruiz E.; Chen X.-M.; Tong M.-L. Redox-Programmable Spin-Crossover Behaviors in a Cationic Framework. J. Am. Chem. Soc. 2022, 144, 14888–14896. 10.1021/jacs.2c06313. 35918175

[ref44] Díaz-Torres R.; Boonprab T.; Gómez-Coca S.; Ruiz E.; Chastanet G.; Harding P.; Harding D. J. Structural and theoretical insights into solvent effects in an iron(iii) SCO complex. Inorg. Chem. Front. 2022, 9, 5317–5326. 10.1039/D2QI01159J.

[ref45] Gómez-Coca S.; Ruiz E. Accurate State Energetics In Spin-Crossover Systems Using Pure Density Functional Theory. Dalton Trans 2024, 10.1039/D4DT00975D. 38953548

[ref46] Kovács P.; Tran F.; Blaha P.; Madsen G. K. H. What is the optimal mGGA exchange functional for solids?. J. Chem. Phys. 2022, 157 (9), 094110 10.1063/5.0098787. 36075720

[ref47] Dema K.; Hooshmand Z.; Pederson M. Electronic and Magnetic Signatures of Low-Lying Spin-Flip Excitonic States of Mn_12_O_12-_Acetate. Polyhedron 2021, 206, 115332 10.1016/j.poly.2021.115332.

[ref48] Fitzhugh H. C.; Furness J. W.; Pederson M. R.; Peralta J. E.; Sun J. Comparative Density Functional Theory Study of Magnetic Exchange Couplings in Dinuclear Transition-Metal Complexes. J. Chem. Theory Comput. 2023, 19, 5760–5772. 10.1021/acs.jctc.3c00336. 37582098 PMC10500985

[ref49] Petrenko A.; Stein M. Environment Effects on Spin States, Properties, and Dynamics from Multi-Level QM/MM Studies. Spin States Biochem. Inorg. Chem. 2015, 327–367. 10.1002/9781118898277.ch14.

[ref50] Harvey J. N.; Poli R.; Smith K. M. Understanding the reactivity of transition metal complexes involving multiple spin states. Coord. Chem. Rev. 2003, 238–239, 347–361. 10.1016/S0010-8545(02)00283-7.

[ref51] Blum V.; Gehrke R.; Hanke F.; Havu P.; Havu V.; Ren X.; Reuter K.; Scheffler M. Ab initio molecular simulations with numeric atom-centered orbitals. Comput. Phys. Commun. 2009, 180, 2175–2196. 10.1016/j.cpc.2009.06.022.

[ref52] Perdew J. P.; Burke K.; Ernzerhof M. Generalized Gradient Approximation Made Simple. Phys. Rev. Lett. 1996, 77, 3865–3868. 10.1103/PhysRevLett.77.3865. 10062328

[ref53] Tkatchenko A.; DiStasio R. A.; Car R.; Scheffler M. Accurate and Efficient Method for Many-Body van der Waals Interactions. Phys. Rev. Lett. 2012, 108, 236402 10.1103/PhysRevLett.108.236402. 23003978

[ref54] van Lenthe E.; Baerends E. J.; Snijders J. G. Relativistic total energy using regular approximations. J. Chem. Phys. 1994, 101, 9783–9792. 10.1063/1.467943.

[ref55] Frisch M. J.; Trucks G. W.; Schlegel H. B.; Scuseria G. E.; Robb M. A.; Cheeseman J. R.; Scalmani G.; Barone V.; Petersson G. A.; Nakatsuji H., Gaussian 16 Rev. C.01, Gaussian: Wallingford, CT; 2016.

[ref56] Becke A. D. Density-Functional Thermochemistry.3. The Role Of Exact Exchange. J. Chem. Phys. 1993, 98, 5648–5652. 10.1063/1.464913.

[ref57] Schafer A.; Huber C.; Ahlrichs R. Fully-optimized contracted gaussian-basis sets of triple zeta valence quality for atoms Li to Kr. J. Chem. Phys. 1994, 100, 5829–5835. 10.1063/1.467146.

[ref58] Grimme S.; Antony J.; Ehrlich S.; Krieg H. A consistent and accurate ab initio parametrization of density functional dispersion correction (DFT-D) for the 94 elements H-Pu. J. Chem. Phys. 2010, 132, 154104 10.1063/1.3382344. 20423165

[ref59] Scott A. P.; Radom L. Harmonic Vibrational Frequencies: An Evaluation of Hartree–Fock, Mo̷ller–Plesset, Quadratic Configuration Interaction, Density Functional Theory, and Semiempirical Scale Factors. J. Phys. Chem. 1996, 100, 16502–16513. 10.1021/jp960976r.

[ref60] Wang S.; Li Y.-J.; Ju F.-F.; Xu W.-T.; Kagesawa K.; Li Y.-H.; Yamashita M.; Huang W. The molecular and supramolecular aspects in mononuclear manganese(iii) Schiff-base spin crossover complexes. Dalton Trans. 2017, 46, 11063–11077. 10.1039/C7DT01718A. 28792029

[ref61] Liu Z.; Liang S.; Di X.; Zhang J. A manganese(III) complex that exhibits spin crossover behavior. Inorg. Chem. Commun. 2008, 11, 783–786. 10.1016/j.inoche.2008.03.030.

[ref62] Hayami S.; Gu Z.-Z.; Shiro M.; Einaga Y.; Fujishima A.; Sato O. First Observation of Light-Induced Excited Spin State Trapping for an Iron(III) Complex. J. Am. Chem. Soc. 2000, 122, 7126–7127. 10.1021/ja001406e.

[ref63] Sim P. G.; Sinn E. 1st Manganese(III) spin crossover and 1st d^4^ crossover - Comment on Cytochrome-Oxidase. J. Am. Chem. Soc. 1981, 103, 241–243. 10.1021/ja00391a067.

[ref64] Guionneau P.; Marchivie M.; Garcia Y.; Howard J. A. K.; Chasseau D. Spin crossover in [Mn^III^(pyrol_3_tren)_2_] probed by high-pressure and low-temperature x-ray diffraction. Phys. Rev. B 2005, 72, 214408 10.1103/PhysRevB.72.214408.

[ref65] Halepoto D. M.; Holt D. G. L.; Larkworthy L. F.; Leigh G. J.; Povey D. C.; Smith G. W. Spin Crossover in Chromium(II) Complexes and the crystal and molecular structure of the high-spin form of bis 1,2-bis(diethylphophino)ethane diiodochromium(II). J. Chem. Soc.-Chem. Commun. 1989, 1322–1323. 10.1039/c39890001322.

[ref66] Pandurangan K.; Gildea B.; Murray C.; Harding C. J.; Mueller-Bunz H.; Morgan G. G. Lattice Effects on the Spin-Crossover Profile of a Mononuclear Manganese(III) Cation. Chem._Eur. J. 2012, 18, 2021–2029. 10.1002/chem.201102820. 22250048

[ref67] Martinho P. N.; Gildea B.; Harris M. M.; Lemma T.; Naik A. D.; Mueller-Bunz H.; Keyes T. E.; Garcia Y.; Morgan G. G. Cooperative Spin Transition in a Mononuclear Manganese(III) Complex. Angew. Chem., Int. Ed. 2012, 51, 12597–12601. 10.1002/anie.201205573. 23129209

[ref68] Walter M. D.; Sofield C. D.; Booth C. H.; Andersen R. A. Spin Equilibria in Monomeric Manganocenes: Solid-State Magnetic and EXAFS Studies. Organometallics 2009, 28, 2005–2019. 10.1021/om800922j.

[ref69] Maeda Y.; Oshio H.; Toriumi K.; Takashima Y. Crystal Structures, Mössbauer spectra and magnetic properties of 2 iron(III) spin-crossover complexes. J. Chem. Soc.-Dalton Trans. 1991, 1227–1235. 10.1039/DT9910001227.

[ref70] Tissot A.; Bertoni R.; Collet E.; Toupet L.; Boillot M.-L. The cooperative spin-state transition of an iron(III) compound Fe^III^(3-MeO-SalEen)_2_ PF_6_: thermal- vs. ultra-fast photo-switching. J. Mater. Chem. 2011, 21, 18347–18353. 10.1039/c1jm14163e.

[ref71] Gallois B.; Real J. A.; Hauw C.; Zarembowitch J. Structural changes associated with the spin transition in [Fe(phen)_2_(NCS)_2_] - A single-crystal X-Ray Investigation. Inorg. Chem. 1990, 29, 1152–1158. 10.1021/ic00331a009.

[ref72] Roux C.; Zarembowitch J.; Gallois B.; Granier T.; Claude R. Toward Ligand-Driven Light-Induced Spin Changing. Influence of the Configuration of 4 Styrylpyridine (stpy) on the Magnetic Properties of [Fe^II^(stpy)_4_(NCS)_2_] Complexes. Crystal Structures of the Spin-Crossover Species [Fe^II^(*trans*-stpy)_4_(NCS)_2_] and of the High-Spin Species [Fe^II^(*cis*-stpy)_4_(NCS)_2_]. Inorg. Chem. 1994, 33, 2273–2279. 10.1021/ic00088a033.

[ref73] Carbonera C.; Kilner C. A.; Letard J.-F.; Halcrow M. A. Anion doping as a probe of cooperativity in the molecular spin-crossover compound FeL_2_ BF_4_ (2) (L = 2,6-di{pyrazol-1-yl}pyridine). Dalton Trans. 2007, 1284–1292. 10.1039/B618480D. 17372643

[ref74] Money V. A.; Radosavljevic Evans I.; Halcrow M. A.; Goeta A. E.; Howard J. A. K. Light induced excited high spin-state trapping in [FeL_2_](BF_4_)_2_ (L = 2,6-di(pyrazol-1-yl)pyridine). Chem. Commun. 2003, 158–159. 10.1039/b210146g. 12611014

[ref75] Real J. A.; Muñoz M. C.; Faus J.; Solans X. Spin crossover in novel dihydrobis(1-pyrazoly)borate H_2_B(pz)_2_ -containing iron(II) complexes. Synthesis, x-ray structure, and magnetic properties of FeL{H_2_B(pz)_2_}_2_ (L = 1,10-phenanthroline and 2,2’-bipyridine). Inorg. Chem. 1997, 36, 3008–3013. 10.1021/ic960965c. 11669951

[ref76] Sheu C.-F.; Chen K.; Chen S.-M.; Wen Y.-S.; Lee G.-H.; Chen J.-M.; Lee J.-F.; Cheng B.-M.; Sheu H.-S.; Yasuda N.; Ozawa Y.; Toriumi K.; Wang Y. Structure and Electronic Configuration of an Iron(II) Complex in a LIESST State: A Pump and Probe Method. Chem._Eur. J. 2009, 15, 2384–2393. 10.1002/chem.200802279. 19142936

[ref77] Figgis B. N.; Kucharski E. S.; White A. H. Crystal-Structure of Bis(2,2’-6’,2’’-terpyridyl)cobalt(II) iodide dihydrate at 295-K and at 120-K. Aust. J. Chem. 1983, 36, 1527–1535. 10.1071/CH9831527.

[ref78] Zarembowitch J.; Kahn O. Magnetic properties of some spin-crossover, high-spin, and low-spin cobalt(II) complexes with Schiff bases derived from 3-formylsalicylic acid. Inorg. Chem. 1984, 23, 589–593. 10.1021/ic00173a020.

[ref79] Gaspar A. B.; Muñoz M. C.; Niel V.; Real J. A. Co^II^(4-terpyridone)_2_ X_2_: A novel cobalt(II) spin crossover system 4-terpyridone = 2,6-bis(2-pyridyl)-4(1H)-pyridone. Inorg. Chem. 2001, 40, 9–10. 10.1021/ic000788m. 11195395

[ref80] Charpin P.; Nierlich M.; Vigner D.; Lance M.; Thuery P.; Zarembowitch J.; D’Yvoire F. Crystal and molecular structure of the spin-crossover complex bis(pyridine) [N,N′-ethylenebis (3-carboxysalicylaldimine)] cobalt (II). J. Crystallog. Spectrosc. Res. 1988, 18, 429–437. 10.1007/BF01195694.

[ref81] Taylor R. A.; Lough A. J.; Lemaire M. T. Spin-crossover in a homoleptic cobalt(II) complex containing a redox-active NNO ligand. J. Mater. Chem. C 2016, 4, 455–459. 10.1039/C5TC03137K.

[ref82] Hayami S.; Nakaya M.; Ohmagari H.; Alao A. S.; Nakamura M.; Ohtani R.; Yamaguchi R.; Kuroda-Sowa T.; Clegg J. K. Spin-crossover behaviors in solvated cobalt(II) compounds. Dalton Trans. 2015, 44, 9345–9348. 10.1039/C4DT03743J. 25599519

[ref83] Holland J. M.; McAllister J. A.; Lu Z.; Kilner C. A.; Thornton-Pett M.; Halcrow M. A. An unusual abrupt thermal spin-state transition in [FeL][BF] [L = 2,6-di(pyrazol-1-yl)pyridine]. Chem. Commun. 2001, 577–578. 10.1039/b100995h.

