# Supplementary material for: Benchmarking Periodic Density Functional Theory Calculations for Spin-State Energies in Spin-Crossover Systems
Source: Inorg Chem. 2024 Jul 8;63(29):13338–45. doi: 10.1021/acs.inorgchem.4c01094 (PMC11270997; doi:10.1021/acs.inorgchem.4c01094)
Supplement: Supplementary file 1 — ic4c01094_si_001.pdf [file ic4c01094_si_001.pdf]

Supporting Information

Benchmarking Periodic Density Functional Theory Calculations for Spin-State Energies in Spin-Crossover Systems

Silvia Gómez-Coca* and Eliseo Ruiz*
Departament de Química Inorgànica i Orgànica and Institut de Recerca de Química Teòrica i Computacional, Universitat de Barcelona,
Diagonal 645, 08028 Barcelona, Spain
emails: silvia.gomez@qi.ub.edu; eliseo.ruiz@qi.ub.edu

Table S1 High- and low-spin states energy differences using discrete molecular models with different grids and basis sets pag. S2

Table S2 k-points in the periodic calculations, and DFT optimized and experimental unit cell volumes pag. S3

Table S3 Experimental critical temperatures and estimated energy differences HS-LS from thermal corrected energies pag. S4

References pag. S5

Table S1. High- and low-spin states energy differences (in kcal/mol) using discrete molecular models (positive values indicate a low-spin ground state) calculated using a meta-GGA functional (r^2SCAN and KTBM24 in bold) using the optimized PBE+MB geometry. The first column corresponds to a tight numerical basis set (t) and a radial grid multiplier value of 2, the second one by improving the grid with a radial multiplier parameter of 4, and the last one by using a *very tight* (vt) basis set with a radial multiplier of 2.

Molecular System	ref.	meta-GGA// PBE+MB t-2		meta-GGA// PBE+MB t-4		meta-GGA// PBE+MB vt-2	
**s1** $[Cr^{II}(L_{s1})_2I_2]$	1	5.7	**5.8**	5.7	**5.8**	5.6	**5.7**
**s2** $[Mn^{III}(L_{s2})]$	2,3	5.0	**4.8**	5.0	**4.8**	5.0	**4.8**
**s3** $[Mn^{III}(L_{s3})]^{+1}$	4	5.5	**5.7**	5.5	**5.7**	5.5	**5.6**
**s4** $[Mn^{III}(L_{s4})]^{+1}$	5	3.1	**3.5**	3.1	**3.6**	3.1	**3.5**
**s5** $[Mn^{II}(L_{s5})_2]$	6	10.1	**9.7**	10.1	**9.7**	10.1	**9.7**
**s6** $[Mn^{II}(L_{s6})_2]$	7	10.4	**11.4**	10.4	**11.4**	10.4	**11.4**
**s7** $[Mn^{II}(L_{s7})_2]$	7	10.3	**11.6**	10.3	**11.6**	10.3	**11.6**
**s8** $[Fe^{III}(L_{s8})]^{+1}$	8	5.3	**7.2**	5.3	**7.2**	5.4	**7.4**
**s9** $[Fe^{III}(L_{s9a})(L_{s9b})_2]^{+1}$	9	12.6	**13.8**	12.6	**13.9**	12.5	**13.9**
**s10** $[Fe^{III}(L_{s10})_2]^{+1}$	10	8.6	**9.5**	8.6	**9.5**	8.7	**9.6**
**s11** $[Fe^{II}(L_{s11})_2(NCS)_2]$	11	7.4	**3.6**	7.4	**3.7**	7.4	**3.7**
**s12** $[Fe(L_{s12})_4(NCS)_2]$	12	9.4	**6.2**	9.4	**6.2**	9.4	**6.2**
**s13** $[Fe^{II}(L_{s13})_2]^{+2}$	13	14.0	**8.2**	14.0	**8.2**	14.0	**8.2**
**s14** $[Fe^{II}(L_{s14a})(L_{s14b})]$	14	12.6	**9.3**	12.6	**9.3**	12.6	**9.3**
**s15** $[Fe^{II}(L_{s15})_2(NCS)_2]$	15	5.8	**2.8**	5.8	**2.8**	5.8	**2.9**
**s16** $[Co^{II}(L_{s16})_2]^{+2}$	16	9.6	**2.3**	9.6	**2.3**	9.5	**2.2**
**s17** $[Co^{II}(L_{s17})(Py)_2]$	17	7.8	**2.8**	7.8	**2.8**	7.5	**2.2**
**s18** $[Co^{II}(L_{s18})_2]^{+2}$	18	8.7	**1.3**	8.7	**1.3**	8.9	**1.8**
**s19** $[Co^{II}(L_{s19})_2]$	19	15.1	**7.1**	15.1	**7.2**	15.2	**7.4**
**s20** $[Co^{II}(L_{s20})_2]^{+2}$	20	10.3	**5.7**	10.3	**5.7**	10.2	**5.7**
**s2new** $[Mn^{III}(L_{s2new})_2]^{+}$	21	4.0	**4.6**	4.0	**4.6**	4.0	**4.6**
**s5new** $[Mn^{III}(L_{s5new})_2]^{+}$	22	1.6	**2.3**	1.6	**2.3**	1.6	**2.3**
**s8new** $[Fe^{III}(L_{s8new})_2]^{+1}$	23	12.2	**9.4**	12.2	**9.4**	12.2	**9.4**

Table S2. K-points employed in the periodic calculations (in parenthesis the split parameters of the reciprocal space), Z is the number of molecules in the unit cell, and in Å^3 PBE-MB optimized and available experimental unit cell volumes for the low- and high-spin state.

**Crystal System**	**ref.**	**k-points**	**Z**	**volume LS**		**volume HS**	
				Opt.	Exp.	Opt.	Exp.
**s1** $[Cr^{II}(L_{s1})_2I_2]$	1	8 (223)	1	680.7		700.4	732.4
**s2new** $[Mn^{III}(L_{s2new})][AsF_6]$	21	2 (211)	4	2516.1	2527.5	2560.9	2626.1
**s3** $[Mn^{III}(L_{s3})][BF_4]$	4	8 (222)	4	3109.0	3042.7	3173.7	3162.6
**s4** $[Mn^{III}(L_{s4})][PF_6]$	5	2 (311)	4	2465.3	2474.7	2507.3	2507.5
**s5new** $[Mn^{III}(L_{s5new})_2]Cl\cdot 3H_2O$	22	4 (221)	2	1213.4	1235.9	1242.0	1248.9
**s6** $[Mn^{II}(L_{s6})_2]$	7	8 (322)	2	747.2	782.8	765.2	
**s7** $[Mn^{II}(L_{s7})_2]$	7	4 (221)	8	4538.5		4721.3	4734.9
**s8new** $[Fe^{III}(L_{s8new})_2][ClO_4]\cdot H_2O$	23	8 (222)	2	1212.0	1217.9	1202.8	1248.9
**s9** $[Fe^{III}(L_{s9a})(L_{s9b})_2][ClO_4]$	9	1 (111)	8	5764.6	5804.5	5966.1	6070.6
**s10** $[Fe^{III}(L_{s10})_2][PF_6]$	10	8 (222)	2	1382.2	1356.4	1404.8	1463.7
**s11** $[Fe^{II}(L_{s11})_2(NCS)_2]$	11	4 (221)	4	2224.8	2219.1	2279.2	2338.2
**s12** $[Fe^{II}(L_{s12})_4(NCS)_2]$	12	4 (122)	4	4593.6		4723.2	4848.8
**s13** $[Fe^{II}(L_{s13})_2][BF_4]_2$	13,24	5 (331)	2	1324.1	1318.2	1350.5	1393.7
**s14** $[Fe^{II}(L_{s14a})(L_{s14b})]$	14	4 (122)	4	2283.3	2356.9	2380.8	2457.1
**s15** $[Fe^{II}(L_{s15})_2(NCS)_2]$	25	4 (212)	2	1184.9	1161.4	1226.6	1244.0
**s16** $[Co^{II}(L_{s16})_2]I_2\cdot 4H_2O$	16	4 (221)	2	1642.7	1506.2	1658.9	1543.1
**s17** $[Co^{II}(L_{s17})(Py)_2]$	17	2 (121)	4	2464.8		2502.2	2568.6
**s18** $[Co^{II}(L_{s18})_2][ClO_4]_2\cdot H_2O$	18, 26	4 (221)	2	1585.3		1599.9	1600.5
**s19** $[Co^{II}(L_{s19})_2]$	19	1 (111)	8	5871.0	5924.4	5891.0	6014.6
**s20** $[Co^{II}(L_{s20})_2][BF_4]_2\cdot H_2O\cdot EtOH$	20	4 (221)	2	1717.9	1701.4	1722.3	

Table S3. Experimental transition temperatures $T_{1/2}$ (in K). Estimation of the difference of the electronic energy (in kcal/mol) to reproduce the experimental $T_{1/2}$ using DFT calculations (B3LYP+GD3 6-311G). Such value can be calculated as the difference of thermal corrections to free energy (thermal corrections to translational, rotational and electronic energy + zeropoint correction + translational, rotational and electronic entropy).

**Molecular System**	**ref.**	**exp. $T_{1/2}$**	**energy HS-LS**
**s1** $[Cr^{II}(L_{s1})_2I_2]$	1	171	1.7
**s2new** $[Mn^{III}(L_{s2new})][AsF_6]$	21	155	2.5
**s3** $[Mn^{III}(L_{s3})][BF_4]$	4	175	2.7
**s4** $[Mn^{III}(L_{s4})][PF_6]$	5	131	2.3
**s5new** $[Mn^{III}(L_{s5new})_2]Cl\cdot 3H_2O$	22	250	2.9
**s6** $[Mn^{II}(L_{s6})_2]$	7	215	6.4
**s7** $[Mn^{II}(L_{s7})_2]$	7	325	9.2
**s8new** $[Fe^{III}(L_{s8new})_2][ClO_4]\cdot H_2O$	23	172	2.7
**s9** $[Fe^{III}(L_{s9a})(L_{s9b})_2][ClO_4]$	9	200	4.0
**s10** $[Fe^{III}(L_{s10})_2][PF_6]$	10	164	3.1
**s11** $[Fe^{II}(L_{s11})_2(NCS)_2]$	11	176.5	4.3
**s12** $[Fe^{II}(L_{s12})_4(NCS)_2]$	12	109	5.4
**s13** $[Fe^{II}(L_{s13})_2][BF_4]_2$	13,24	256	5.3
**s14** $[Fe^{II}(L_{s14a})(L_{s14b})]$	14	160	4.0
**s15** $[Fe^{II}(L_{s15})_2(NCS)_2]$	25	118	1.7
**s16** $[Co^{II}(L_{s16})_2]I_2\cdot 4H_2O$	16	200	2.0
**s17** $[Co^{II}(L_{s17})(Py)_2]$	17	121	1.2
**s18** $[Co^{II}(L_{s18})_2][ClO_4]_2\cdot H_2O$	18, 26	172.4	1.6
**s19** $[Co^{II}(L_{s19})_2]$	19	150	1.5
**s20** $[Co^{II}(L_{s20})_2][BF_4]_2\cdot H_2O\cdot EtOH$	20	250	1.7

References

(1) Halepoto, D. M.; Holt, D. G. L.; Larkworthy, L. F.; Leigh, G. J.; Povey, D. C.; Smith, G. W. Spin Crossover in Chromium(II) Complexes and the crystal and molecular structure of the high-spin form of bis 1,2-bis(diethylphophino)ethane diiodochromium(II). *J. Chem. Soc.-Chem. Commun.* **1989**, (18), 1322-1323.

(2) Sim, P. G.; Sinn, E. 1st Manganese(III) spin crossover and 1st d^4 crossover - Comment on Cytochrome-Oxidase. *J. Am. Chem. Soc.* **1981**, *103* (1), 241-243.

(3) Guionneau, P.; Marchivie, M.; Garcia, Y.; Howard, J. A. K.; Chasseau, D. Spin crossover in [Mn^{III}(pyrol$_3$tren)] probed by high-pressure and low-temperature x-ray diffraction. *Phys. Rev. B* **2005**, *72* (21), 214408.

(4) Pandurangan, K.; Gildea, B.; Murray, C.; Harding, C. J.; Mueller-Bunz, H.; Morgan, G. G. Lattice Effects on the Spin-Crossover Profile of a Mononuclear Manganese(III) Cation. *Chem. Eur. J.* **2012**, *18* (7), 2021-2029.

(5) Martinho, P. N.; Gildea, B.; Harris, M. M.; Lemma, T.; Naik, A. D.; Mueller-Bunz, H.; Keyes, T. E.; Garcia, Y.; Morgan, G. G. Cooperative Spin Transition in a Mononuclear Manganese(III) Complex. *Angew. Chem. Int. Ed.* **2012**, *51* (50), 12597-12601.

(6) Switzer, M. E.; Wang, R.; Rettig, M. F.; Maki, A. H. Electronic ground states of manganocene and 1,1'-dimethylmanganocene. *J. Am. Chem. Soc.* **1974**, *96* (25), 7669-7674.

(7) Walter, M. D.; Sofield, C. D.; Booth, C. H.; Andersen, R. A. Spin Equilibria in Monomeric Manganocenes: Solid-State Magnetic and EXAFS Studies. *Organometallics* **2009**, *28* (7), 2005-2019.

(8) Dose, E. V.; Murphy, K. M. M.; Wilson, L. J. Synthesis and spin-state studies in solution of γ-substituted tris(β-diketonato) iron(III) complexes and their spin-equilibrium β-ketoimine analogues derived from triethylenetetramine. *Inorg. Chem.* **1976**, *15* (11), 2622-2630.

(9) Maeda, Y.; Oshio, H.; Toriumi, K.; Takashima, Y. Crystal Structures, Mössbauer spectra and magnetic properties of 2 iron(III) spin-crossover complexes. *J. Chem. Soc.-Dalton Trans.* **1991**, (5), 1227-1235.

(10) Tissot, A.; Bertoni, R.; Collet, E.; Toupet, L.; Boillot, M.-L. The cooperative spin-state transition of an iron(III) compound Fe^{III}(3-MeO-SalEen)$_2$(PF_6): thermal- vs. ultra-fast photo-switching. *J. Mater. Chem.* **2011**, *21* (45), 18347-18353.

(11) Gallois, B.; Real, J. A.; Hauw, C.; Zarembowitch, J. Structural changes associated with the spin transition in [Fe(Phen)$_2$(NCS)$_2$] - A single-crystal X-Ray Investigation. *Inorg. Chem.* **1990**, *29* (6), 1152-1158.

(12) Roux, C.; Zarembowitch, J.; Gallois, B.; Granier, T.; Claude, R. Toward Ligand-Driven Light-Induced Spin Changing. Influence of the Configuration of 4 Styrylpyridine (stpy) on the Magnetic Properties of [Fe^{II}(stpy)$_4$(NCS)$_2$] Complexes. Crystal Structures of the Spin-Crossover Species [Fe^{II}(*trans*-stpy)$_4$(NCS)$_2$] and of the High-Spin Species [Fe^{II}(*cis*-stpy)$_4$(NCS)$_2$] *Inorg. Chem.* **1994**, *33* (10), 2273-2279.

(13) Carbonera, C.; Kilner, C. A.; Letard, J.-F.; Halcrow, M. A. Anion doping as a probe of cooperativity in the molecular spin-crossover compound $FeL_2(BF_4)_2$ (L=2,6-di{pyrazol-1-yl}pyridine). *Dalton Trans.* **2007**, (13), 1284-1292.

(14) Real, J. A.; Muñoz, M. C.; Faus, J.; Solans, X. Spin crossover in novel dihydrobis(1-pyrazoly)borate H_2Bpz_2 -containing iron(II) complexes. Synthesis, x-ray structure, and magnetic properties of FeL{ H_2Bpz_2 }$_2$ (L=1,10-phenanthroline and 2,2'-bipyridine). *Inorg. Chem.* **1997**, *36* (14), 3008-3013.

(15) Niel, V.; Gaspar, A. B.; Muñoz, M. C.; Abarca, B.; Ballesteros, R.; Real, J. A. Spin crossover behavior in the iron(II)-2-pyridyl 1,2,3 triazolo 1,5-a pyridine system: X-ray structure, calorimetric, magnetic, and photomagnetic studies. *Inorg. Chem.* **2003**, *42* (15), 4782-4788.

(16) Figgis, B. N.; Kucharski, E. S.; White, A. H. Crystal-Structure of Bis(2,2'-6',2''-terpyridyl)cobalt(II) iodide dihydrate at 295-K and at 120-K. *Aust. J. Chem.* **1983**, *36* (8), 1527-1535.

(17) Zarembowitch, J.; Kahn, O. Magnetic properties of some spin-crossover, high-spin, and low-spin cobalt(II) complexes with Schiff bases derived from 3-formylsalicylic acid. *Inorg. Chem.* **1984**, *23* (5), 589-593.

(18) Gaspar, A. B.; Muñoz, M. C.; Niel, V.; Real, J. A. Co^{II}(4-terpyridone)(2) X-2: A novel cobalt(II) spin crossover system 4-terpyridone=2,6-bis(2-pyridyl)-4(1H)-pyridone. *Inorg. Chem.* **2001**, *40* (1), 9-10.

(19) Taylor, R. A.; Lough, A. J.; Lemaire, M. T. Spin-crossover in a homoleptic cobalt(II) complex containing a redox-active NNO ligand. *J. Mater. Chem. C* **2016**, *4* (3), 455-459.

(20) Hayami, S.; Nakaya, M.; Ohmagari, H.; Alao, A. S.; Nakamura, M.; Ohtani, R.; Yamaguchi, R.; Kuroda-Sowa, T.; Clegg, J. K. Spin-crossover behaviors in solvated cobalt(II) compounds. *Dalton Trans.* **2015**, *44* (20), 9345-9348.

(21) Wang, S.; Li, Y.-J.; Ju, F.-F.; Xu, W.-T.; Kagesawa, K.; Li, Y.-H.; Yamashita, M.; Huang, W. The molecular and supramolecular aspects in mononuclear manganese(iii) Schiff-base spin crossover complexes. *Dalton Trans.* **2017**, *46* (33), 11063-11077.

(22) Liu, Z.; Liang, S.; Di, X.; Zhang, J. A manganese(III) complex that exhibits spin crossover behavior. *Inorg. Chem. Commun.* **2008**, *11* (7), 783-786.

(23) Hayami, S.; Gu, Z.-z.; Shiro, M.; Einaga, Y.; Fujishima, A.; Sato, O. First Observation of Light-Induced Excited Spin State Trapping for an Iron(III) Complex. *J. Am. Chem. Soc.* **2000**, *122* (29), 7126-7127.

(24) Money, V. A.; Radosavljevic Evans, I.; Halcrow, M. A.; Goeta, A. E.; Howard, J. A. K. Light induced excited high spin-state trapping in $[FeL_2](BF_4)_2$ (L = 2,6-di(pyrazol-1-yl)pyridine). *Chem. Commun.* **2003**, (1), 158-159.

(25) Sheu, C.-F.; Chen, K.; Chen, S.-M.; Wen, Y.-S.; Lee, G.-H.; Chen, J.-M.; Lee, J.-F.; Cheng, B.-M.; Sheu, H.-S.; Yasuda, N.; et al. Structure and Electronic Configuration of an Iron(II) Complex in a LIESST State: A Pump and Probe Method. *Chem. Eur. J.* **2009**, *15* (10), 2384-2393.

(26) Charpin, P.; Nierlich, M.; Vigner, D.; Lance, M.; Thuery, P.; Zarembowitch, J.; D'Yvoire, F. Crystal and molecular structure of the spin-crossover complex bis(pyridine) [N,N′ -ethylenebis (3-carboxysalicylaldimine)] cobalt (II). *J Crystallogr. Spectrosc. Res.* **1988**, *18* (4), 429-437.
